# Supplementary material for: Comparison of machine learning methods for genomic prediction of selected Arabidopsis thaliana traits
Source: PLoS One. 2024 Aug 28;19(8):e0308962. doi: 10.1371/journal.pone.0308962 (PMC11355539; doi:10.1371/journal.pone.0308962)
Supplement: S1 File — (DOCX) [file pone.0308962.s002.docx]

**S1 Grid Search Parameters.**

**Feed-forward neural network**: Number of units, learning rate, optimizer, batch size,

network shape, L1/L2 regularization, dropout rate, number of hidden layers, epochs,

kernel initializer, activation function

**Convolutional neural network**: Number of units, learning rate, optimizer, batch size,

network shape, L1/L2 regularization, dropout rate, number of hidden layers, epochs,

kernel initializer, activation function, filters, kernel, pool, strides

**SVM**: C, gamma, epsilon, loss, kernel, degree, cache size, tolerance

**LASSO**: Alpha, selection, tolerance, maximum iterations

**Ridge regression:** Alpha, tolerance

**Random forests:** Number of estimators, maximum depth, maximum number of

features, bootstrapping, maximum leaf nodes, maximum number of samples, minimum

samples to split, minimum leaf samples, maximum leaf nodes
