# Supplementary material for: Comparison of machine learning methods for genomic prediction of selected Arabidopsis thaliana traits
Source: PLoS One. 2024 Aug 28;19(8):e0308962. doi: 10.1371/journal.pone.0308962 (PMC11355539; doi:10.1371/journal.pone.0308962)
Supplement: Attachment — Submitted filename: PONE-D-23-39918_reviewer.pdf [file pone.0308962.s003.pdf]

PLOS ONE

Comparison of machine learning methods for genomic prediction of Arabidopsis thaliana traits

--Manuscript Draft--

**Manuscript Number:**	PONE-D-23-39918
**Article Type:**	Research Article
**Full Title:**	Comparison of machine learning methods for genomic prediction of Arabidopsis thaliana traits
**Short Title:**	Comparison of machine learning methods for genomic prediction
**Corresponding Author:**	Ciaran Michael Kelly, Ph.D.<br>RCSI University of Medicine and Health Sciences: RCSI Dublin<br>Dublin, IRELAND
**Keywords:**	Genomic Prediction; Machine Learning; Arabidopsis Thaliana; Epistasis; Genomics
**Abstract:**	We present a comparison of machine learning methods for the prediction of several quantitative traits in Arabidopsis thaliana. High prediction accuracies were achieved on individuals grown under standardized laboratory conditions from the 1001 Arabidopsis Genomes Project. An existing body of evidence suggests that linear models may be impeded by their inability to make use of non-additive effects to explain phenotypic variation at the population level. The results presented here use a nested cross-validation approach that confirms that some machine learning methods have the ability to outperform linear prediction models, with the optimal model dependent on availability of training data and genetic architecture of the trait in question. Generally, the neural network class of predictors was observed to be the most accurate and robust for traits with high heritability. Further understanding of the genetic architecture of each trait, larger sample sizes and the development of models of greater complexity may lead to future improvements in prediction accuracy.
**Order of Authors:**	Ciaran Michael Kelly, Ph.D.
	Russell Lewis McLaughlin, Ph.D.
**Additional Information:**	
**Question**	**Response**
**Financial Disclosure**<br><br>Enter a financial disclosure statement that describes the sources of funding for the work included in this submission. Review the submission guidelines for detailed requirements. View published research articles from *PLOS ONE* for specific examples.<br><br><br>This statement is required for submission and **will appear in the published article** if the submission is accepted. Please make sure it is accurate.	Yes

Powered by Editorial Manager® and ProduXion Manager® from Aries Systems Corporation

**Funded studies**<br>Enter a statement with the following details:<br>• Initials of the authors who received each award<br>• Grant numbers awarded to each author<br>• The full name of each funder<br>• URL of each funder website<br>• Did the sponsors or funders play any role in the study design, data collection and analysis, decision to publish, or preparation of the manuscript?<br><br>Did you receive funding for this work?	
Please add funding details.<br>as follow-up to "**Financial Disclosure**<br><br>Enter a financial disclosure statement that describes the sources of funding for the work included in this submission. Review the submission guidelines for detailed requirements. View published research articles from *PLOS ONE* for specific examples.<br><br>This statement is required for submission and **will appear in the published article** if the submission is accepted. Please make sure it is accurate.<br><br>**Funded studies**<br>Enter a statement with the following details:<br>• Initials of the authors who received each award<br>• Grant numbers awarded to each author<br>• The full name of each funder<br>• URL of each funder website<br>• Did the sponsors or funders play any role in the study design, data collection and analysis, decision to publish, or preparation of the manuscript?<br><br>Did you receive funding for this work?"	This study was supported by Science Foundation Ireland (17/CDA/4737). Russell McLaughlin also receives support from the MND Association (879-791) and Science Foundation Ireland<br>(16/RC/3948).
Please select the country of your main research funder (please select carefully as in some cases this is used in fee calculation).	IRELAND - IE

as follow-up to "**Financial Disclosure**<br><br>Enter a financial disclosure statement that describes the sources of funding for the work included in this submission. Review the submission guidelines for detailed requirements. View published research articles from *PLOS ONE* for specific examples.<br><br>This statement is required for submission and **will appear in the published article** if the submission is accepted. Please make sure it is accurate.<br><br>**Funded studies**<br>Enter a statement with the following details:<br>• Initials of the authors who received each award<br>• Grant numbers awarded to each author<br>• The full name of each funder<br>• URL of each funder website<br>• Did the sponsors or funders play any role in the study design, data collection and analysis, decision to publish, or preparation of the manuscript?<br><br>Did you receive funding for this work?"	
**Competing Interests**<br><br>Use the instructions below to enter a competing interest statement for this submission. On behalf of all authors, disclose any competing interests that could be perceived to bias this work—acknowledging all financial support and any other relevant financial or non-financial competing interests.<br><br>This statement is required for submission and **will appear in the published article** if the submission is accepted. Please make sure it is accurate and that any funding sources listed in your Funding Information later in the submission form are also declared in your Financial Disclosure statement.	The authors have declared that no competing interests exist.

View published research articles from *PLOS ONE* for specific examples.<br><br>**NO authors have competing interests**<br><br>Enter: *The authors have declared that no competing interests exist.*<br><br>**Authors with competing interests**<br><br>Enter competing interest details beginning with this statement:<br><br>*I have read the journal's policy and the authors of this manuscript have the following competing interests: [insert competing interests here]*<br><br>* typeset	
**Ethics Statement**<br><br>Enter an ethics statement for this submission. This statement is required if the study involved:<br><br>• Human participants<br>• Human specimens or tissue<br>• Vertebrate animals or cephalopods<br>• Vertebrate embryos or tissues<br>• Field research<br><br>Write "N/A" if the submission does not require an ethics statement.<br><br>General guidance is provided below. Consult the submission guidelines for detailed instructions. **Make sure that all information entered here is included in the Methods section of the manuscript.**	N/A

Powered by Editorial Manager® and ProduXion Manager® from Aries Systems Corporation

**Format for specific study types**<br><br>**Human Subject Research (involving human participants and/or tissue)**<br>• Give the name of the institutional review board or ethics committee that approved the study<br>• Include the approval number and/or a statement indicating approval of this research<br>• Indicate the form of consent obtained (written/oral) or the reason that consent was not obtained (e.g. the data were analyzed anonymously)<br><br>**Animal Research (involving vertebrate animals, embryos or tissues)**<br>• Provide the name of the Institutional Animal Care and Use Committee (IACUC) or other relevant ethics board that reviewed the study protocol, and indicate whether they approved this research or granted a formal waiver of ethical approval<br>• Include an approval number if one was obtained<br>• If the study involved *non-human primates*, add *additional details* about animal welfare and steps taken to ameliorate suffering<br>• If anesthesia, euthanasia, or any kind of animal sacrifice is part of the study, include briefly which substances and/or methods were applied<br><br>**Field Research**<br><br>Include the following details if this study involves the collection of plant, animal, or other materials from a natural setting:<br>• Field permit number<br>• Name of the institution or relevant body that granted permission	
**Data Availability**<br><br>Authors are required to make all data underlying the findings described fully available, without restriction, and from the time of publication. PLOS allows rare exceptions to address legal and ethical concerns. See the PLOS Data Policy and FAQ for detailed information.	Yes - all data are fully available without restriction

Powered by Editorial Manager® and ProduXion Manager® from Aries Systems Corporation

A Data Availability Statement describing where the data can be found is required at submission. Your answers to this question constitute the Data Availability Statement and **will be published in the article**, if accepted.<br><br>**Important:** Stating 'data available on request from the author' is not sufficient. If your data are only available upon request, select 'No' for the first question and explain your exceptional situation in the text box.<br><br>Do the authors confirm that all data underlying the findings described in their manuscript are fully available without restriction?	
**Describe where the data may be found in full sentences. If you are copying our sample text, replace any instances of XXX with the appropriate details.**<br><br>• If the data are **held or will be held in a public repository**, include URLs, accession numbers or DOIs. If this information will only be available after acceptance, indicate this by ticking the box below. For example: *All XXX files are available from the XXX database (accession number(s) XXX, XXX.).*<br>• If the data are all contained **within the manuscript and/or Supporting Information files**, enter the following: *All relevant data are within the manuscript and its Supporting Information files.*<br>• If neither of these applies but you are able to provide **details of access elsewhere**, with or without limitations, please do so. For example:<br><br>*Data cannot be shared publicly because of [XXX]. Data are available from the XXX Institutional Data Access / Ethics Committee (contact via XXX) for researchers who meet the criteria for access to confidential data.*<br><br>*The data underlying the results presented in the study are available from (include the name of the third party*	All genotypic and phenotypic data is available from the 1001 Arabidopsis Project (https://1001genomes.org/data-center.html)<br><br>All optimizing and statistical code is available from Github (https://github.com/ciaranoceallaigh96/arabidopsis_ml/)

and contact information or URL).<br>• This text is appropriate if the data are owned by a third party and authors do not have permission to share the data.<br><br>* typeset	
Additional data availability information:	

LaTeX PDF Click here to access/download;Manuscript;plos_one_draft_v1.pdf

Comparison of machine learning methods for genomic prediction of *Arabidopsis thaliana* traits

Ciaran Kelly[1*¤], Russell McLaughlin[1]

1 Smurfit Institute of Genetics, Trinity College Dublin, Dublin, Ireland

¤Current Address: School of Pharmacy and Biomolecular Sciences, RCSI University of Medicine and Health Sciences, Dublin, Ireland

* oceallc@tcd.ie

Abstract

We present a comparison of machine learning methods for the prediction of several quantitative traits in *Arabidopsis thaliana*. High prediction accuracies were achieved on individuals grown under standardized laboratory conditions from the *1001 Arabidopsis Genomes Project*. An existing body of evidence suggests that linear models may be impeded by their inability to make use of non-additive effects to explain phenotypic variation at the population level. The results presented here use a nested cross-validation approach that confirms that some machine learning methods have the ability to outperform linear prediction models, with the optimal model dependent on availability of training data and genetic architecture of the trait in question. Generally, the neural network class of predictors was observed to be the most accurate and robust for traits with high heritability. Further understanding of the genetic architecture of each trait, larger sample sizes and the development of models of greater complexity may lead to future improvements in prediction accuracy.

Introduction

Genomic prediction

A major goal in the field of genetics is to make use of genotypic information to predict phenotypic values at both the individual and population level. When considering a population, the amount of variance that can be explained by genetics is bounded by the broad-sense heritability (H^2). This parameter, the ratio of genotypic to phenotypic variance, is not fixed in time or space, and can vary substantially between traits [1].

In practice, genomic prediction has been achieved largely through the development of linear models, often with great success [2, 3]. However, linear models are naturally limited by their inability to incorporate non-linear statistical effects and are instead bounded by the narrow-sense heritability (h^2) which is defined as the proportion of additive genetic variance to overall phenotypic variance. Thus, linear models may not account for non-additive variation, such as that which could arise from complex non-linear interactions between genetic variants across the genome, i.e. statistical epistasis [4].

It must be noted that these linear approaches do not correspond to only modelling additive gene action at the molecular level. Importantly, non-additive biological effects can be partly subsumed into additive variance and indeed be exploited for prediction

purposes [1]. It is for this reason that many negate the importance of non-linear models for improving performance or accounting for “missing heritability”, that is the difference that exists between the phenotypic variance estimated to be explainable by genetic variation and the current performance of the models that have thus far been developed [5–7].

Nevertheless, a substantial amount of variation may not be captured by additive variance and it is not necessarily the case that the standard parameterization of variance into additive and non-additive effects is always the ideal framework for approaching a given genomic prediction task [8–10].

The optimal prediction method depends on the actual genetic architecture of the trait, even when the magnitude of non-additive variance is significantly less than the additive variance. It cannot be assumed *a priori* that linear models will be the most favourable prediction approach for any given trait of interest.

Notwithstanding these limitations, linear prediction models are generally simple to build, interpret, and transfer across datasets. Indeed, they have seen much success over the last few decades in predicting individual plant phenotypes and in genomic selection using single nucleotide polymorphism (SNP) data [3, 11]. This approach generally involves measuring the associations between genetic markers and the trait of interest and, simultaneously or subsequently, generating a polygenic prediction model from the subsets of those highlighted quantatative trait loci (QTLs) that are most associated. Common association and prediction methods include genome-wide association studies (GWAS) and genomic best linear unbiased prediction (gBLUP).

The gBLUP method makes use of a linear mixed model, where SNPs are modelled as [illegible]d effects, to evaluate all possible QTLs in the genome, with the assumption that effect sizes are small and normally distributed. gBLUP was introduced as an extension of the traditional best linear unbiased prediction (BLUP) through the incorporation of a genomic relationship matrix (GRM) rather than the previous reliance on pedigree information in accounting for covariance between relatives in the population [12, 13]. The GRM consists of the realized proportion of shared genome for each pair of individuals within the matrix. gBLUP is generally more accurate than BLUP as it relies on the actual relatedness between individuals (even distantly so) rather than their assumed relationship. Any new individual with known genotype and unknown phenotype can get a reasonable estimate for the trait value of interest using this method. It is a standard method of choice in animal and plant association/prediction tasks, while also being applicable to human data [14].

Recently, the field of machine learning has come to the fore in offering promising methods for achieving higher polygenic prediction accuracies, as they have greater flexibility to model the complexity that may underlie the genetic proportion of phenotypic variance. It is possible that some machine learning methods can make progress in accounting for some of the missing heritability that remains to be explained.

Machine learning

Machine learning refers to a set of algorithms that are trained to detect and exploit complex patterns in data that can then be used as predictors on separate unseen test datasets. For genomic prediction, the training dataset generally consists of individual-level genotype inputs and phenotype value outputs. As the training individuals’ trait values are labelled, this is a supervised class of prediction problem [15].

Although more simple models may provide causal insights into how input variables relate to the output (i.e. genotype to phenotype), the appeal of some machine learning families lies in their greater flexibility to model complexity, such as that which may arise from the genetic architecture of a trait.

In doing so, it is important to note that not all machine learning methods are non-linear, such as regularised regression models (Ridge and LASSO) or linear support vector machines (SVMs). However, these approaches may, in some instances, still be more suitable for the genetic architecture underlying certain traits (e.g. via effective regularisation and feature selection) and the resulting models may provide a more appropriate framework to capture some non-additive biological action in the marginal effects of risk alleles.

When training a machine learning model, many hyperparameters of the chosen model type may need to be optimized, depending on the specific machine learning method in question. This can be achieved through searching various combinations of these hyperparameters, with time and computational power usually being limiting factors. As a result of these complex optimization techniques, the final models are often not readily interpretable because they can involve complicated high-dimensional non-linearities.

Several previous studies have been published on the use of machine learning for genomic prediction in plants [16–19]. In general, some machine learning methods are reported to outperform linear models, although not universally so, and the magnitude of that benefit is variable [20]. Importantly, a major concern in the development of machine learning models is overfitting of the data during training and testing, with lower performance being observed on independent datasets. Methods for determining model robustness should therefore be a key priority for the field.

There exist multiple machine learning methods that can be used for supervised prediction tasks such as genomic prediction. Support vector machines, neural networks, random forests, LASSO regression and ridge regression were chosen for this analysis. Baseline gBLUP models were also developed in order to gauge the accuracy and robustness of the prediction models.

Performance

The coefficient of determination is sometimes used in measuring the performance of a genomic prediction model. However, the interpretation of R^2 as the amount of variance explained by the model does not hold for non-linear models, and caution is warranted when employing this metric on such models [21–23].

This is unfortunate, as the R^2 lies on the same scale as the heritability and would be useful in benchmarking performance against the maximum prediction theoretically possible through using genomic information. Instead, an alternative metric that is often employed in plant and animal genetics is Pearson's correlation coefficient (ρ) between predictions and measurements [24].

In order to estimate predictive performance on outside datasets, a hold-out set or a cross-validation strategy is usually applied to machine learning procedures [15]. k-fold cross-validation is generally the preferred method of choice, and involves splitting the data k times, each time resulting in an independent training and validation set of individuals. This way, the model is trained and evaluated k times, and gives a more accurate estimate of the prediction capability on independent test data. Overfitting is avoided, as would happen in the naive approach whereby a model is trained and then evaluated on the entire dataset. An additional benefit is that all of the data are utilized for training purposes, unlike the hold-out method, resulting in greater power. In the case where hyperparameters are also being selected, a nested cross-validation procedure is required in order to avoid information leak (and thus an overly-optimistic result) from the validation sets [25–27]. The nested approach involves further performing an n-fold cross-validation on each of the k training sets, resulting in inner training and validation sets alongside the original outer sets (see Fig. 1 for a stepwise explanation of the procedure). The best hyperparameter combinations are chosen within each inner n split

and then applied independently on each outer training set before being evaluated on the final k outer validation sets.

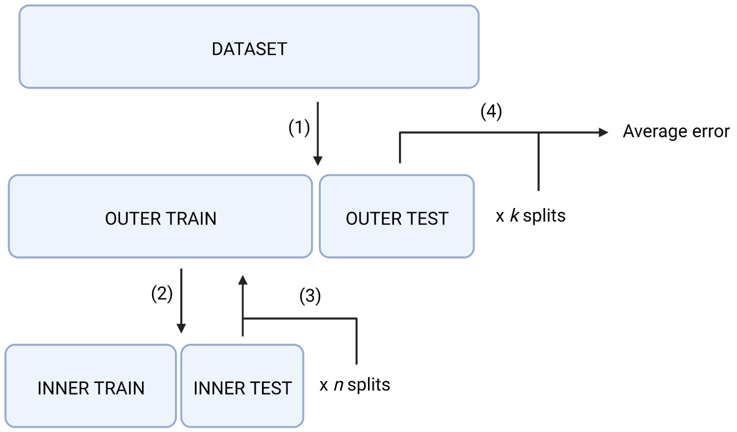

F[illegible]. A nested cross-validation procedure. **(1)** Split entire dataset k times into outer train and test sets. **(2)** Split each k outer train set into n inner train and test sets. **(3)** By training on each inner train set, find the overall best hyperparameter combinations across the n test sets and then apply this to train the corresponding outer train set. **(4)** Evaluate final k trained models on their corresponding test sets and average the errors.

In the non-nested approach, one would end up choosing the best hyperparameter values from repeated testing on the k validation sets, resulting in an optimistic estimate of the actual predictive performance. When the cross-validation is nested, the hyperparameter values are only configured within the inner n loops and so the error when applied to the outer validation sets should result in a more realistic estimate of the performance on an independent dataset.

This is not commonly implemented in the genomic prediction literature, despite the risks that simple cross-validation entails [28]. Thus, in this paper we chose to employ a fully nested cross-validation hyperparameter search in the absence of an available independent dataset. The resulting estimated performance should be relatively free from bias introduced by both hyperparameter tuning and model training.

Materials and methods

Data

Open-access *Arabidopsis* SNP data was accessed from the 1001 *Arabidopsis* Project across a variety of phenotypes [29]. This dense dataset contains 10,707,430 SNPs across all four chromosomes. Individuals are highly inbred resulting in homozygosity at every position in the genome.

"Time to first flowering" was chosen as the main trait of interest as the genetics of the trait has been extensively studied and had the largest sample size of both genotype and phenotype information. This trait was studied across two laboratory temperatures: 10°C and 16°C. The other traits examined are "seed yield" (dried weight in grams) and "leaf area" (in centimeters squared). All individuals were grown under standard laboratory procedures and conditions, constraining the environmental component of trait heritability.

Heritability Estimation

GREML analysis was used to estimate the additive SNP-based heritability (h^2_{SNP}) for each trait using GCTA software version 1.92.0 [14, 30].

The GRM used for this analysis was built using a set of 585,375 pruned SNPs. The pruning procedure was conducted in PLINK2 and used a 250kb window, 5 bp step-size and an r^2 threshold of 0.05 [31]. A minimum minor allele count of 6 was chosen for SNP inclusion.

Experimental Approach

Using genomic data from the samples of *Arabidopsis*, the objective of this analysis was to compare the relative performance of the various machine learning methods to the baseline linear models. As well as gBLUP, we assessed the performance of least absolute shrinkage and selection operator (LASSO) regression, ridge regression, random forests, support vector machines (linear and non-linear), feed-forward neural networks and convolutional neural networks. A brief explanation of each of these approaches is provided in supplementary information S1.

The influence of feature set size and effect of prior linear feature selection were also investigated in this analysis. Prediction accuracies of machine learning methods were compared against gBLUP prediction as a baseline, and for each trait the SNP-based heritability was also calculated to gauge the overall performance of the models.

Feature Selection

Two feature selection strategies were adopted in order to select the input SNP set from the dense genotype dataset, with feature selection performed independently on all inner and outer loops of this analysis. The first strategy was to use a GWAS to identify the SNPs most linearly associated with the trait. The results were subsequently clumped to generate the set of top SNPs free from strong linkage disequilibrium (LD).

GWASs were performed on the various *Arabidopsis* phenotypes using PLINK2 GLM association [32]. A minimum allele count of 20 was used, and the resulting SNPs were clumped using the following parameters: significance threshold of 0.05, secondary significance threshold of 0.1, LD threshold of 0.05 and distance threshold of 250kb.

The second approach was to omit an initial GWAS and select SNPs randomly from across the genome. SNPs were first pruned down to an LD-independent set using a 250kb window, 5bp step-size and an r^2 threshold of 0.05. A minimum allele count of 6 was used.

Model Optimization

In order to implement nested-cross validation a modified version of the Python NestedCV package was used (https://github.com/casperbh96/Nested-Cross-Validation), allowing for logging and plotting of inner loop test results and pre-specified random cross-validation splits. Cross-validation splits were made with pre-shuffling using Scikit-learn's KFold function [33].

gBLUP prediction was performed in GCTA using the previously described GRMs and the subset of feature selected SNPs [14, 34].

All regression, tree and kernel methods were performed using the Scikit-learn library in Python3. Neural networks were implemented using Keras on a TensorFlow backend [35, 36]. The mean absolute error loss metric was chosen for all machine learning algorithms to prioritize robustness [15].

Genotypes were loaded in as binary variables along with corresponding phenotypes. Phenotype values were standardized and scaled based on the training set prior to learning. Random grid searching and manual evaluation on the inner loops was implemented to optimize the hyperparameters.

Performance Assessment

In order to compare predictivity across all methods used, Pearson's correlation coefficient on the test set was calculated.

A multiple-comparison Dunnett test was used in order to test for significant differences in performance between the machine learning models and the baseline gBLUP model [37, 38]. This was implemented in a non-parametric and small sample size design using the nparcomp and mlt packages in R-3.6.3 [39–41].

Results

Heritability Estimation

The SNP-based narrow-sense heritabilities for each trait are shown in Table 1. For the flowering time traits, the values are relatively high, which may be explained by the fact that [illegible] genetic variance was constrained by the laboratory conditions under which the plants grew. Given that nearly all variation in flowering time is captured by genetic effects, it is likely that genomic prediction models could be useful in accounting for trait variance in the population.

Table 1. SNP-based narrow-sense trait heritabilities (h^2_{SNP}) of four *Arabidopsis thaliana* traits.

Phenotype	N	h^2_{SNP}	s.e.
Flowering Time (10°C)	1058	0.943	0.057
Flowering Time (16°C)	1021	0.904	0.057
Seed Yield	384	0.236	0.101
Leaf Area	445	0.300	0.093

For the other two traits, seed yield and leaf area, the heritabilities were lower. It is possible that their smaller sample size contributed to this result, as well as a larger element of random or developmental variation underlying the traits.

Genomic Prediction

The results for the flowering traits can be seen in Figs. 2 and 3. Some machine learning families consistently outperform the gBLUP method, often significantly so. Importantly, this was not true for all families and shows that the linear gBLUP serves as as important baseline upon which to improve. For context in terms of heritability and of the amount of variation being explained by the models, the R^2 of the gBLUP models are given in the figure legends.

Feed-forward neural networks were the best method on average in terms of overall ranking, as well as the model that was significantly improved from the baseline the most times. For this reason, they could be seen as the machine learning family that has the most potential to improve over gBLUP. Although neural networks tend to have a lot of tuned hyperparameters, and are thus prone to overfitting, this was not seen to a detrimental extent in the outer loops of the nested cross-validation. Ridge regression, which is a linear model, also performed generally quite well.

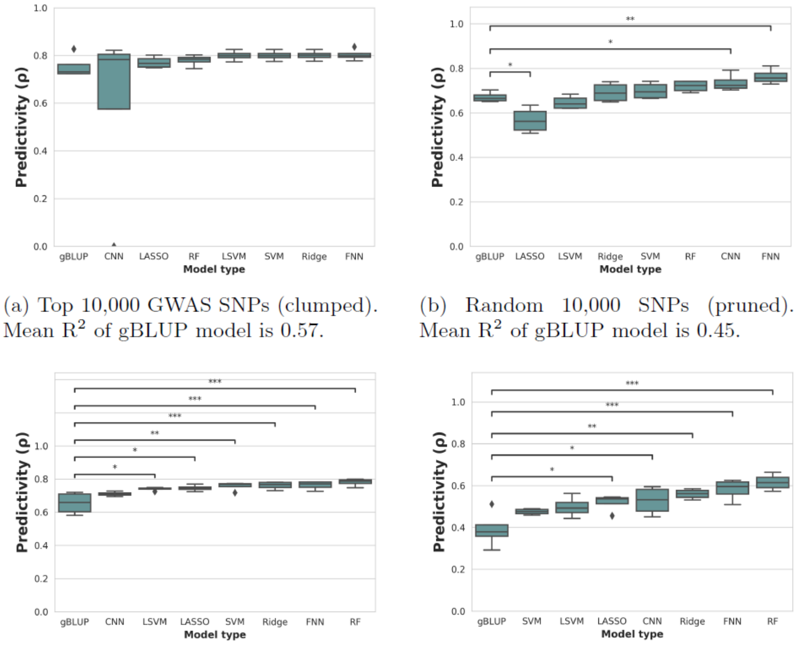

(a) Top 10,000 GWAS SNPs (clumped). Mean R^2 of gBLUP model is 0.57.

(b) Random 10,000 SNPs (pruned). Mean R^2 of gBLUP model is 0.45.

(c) Top 1,000 GWAS SNPs (clumped). Mean R^2 of gBLUP model is 0.43.

(d) Random 1,000 SNPs (pruned). Mean R^2 o[illegible]LUP model is 0.16.

Fig 2. Nested cross-validation results for flowering time (10°C). Models are sorted by mean performance. Dunnett test p-value annotations: $*, p < 0.05$; $**, p < 0.01$; $***, p < 0.001$. gBLUP, genomic best linear unbiased prediction; CNN, convolutional neural network; RF, random forests; LASSO, least absolute shrinkage and selection operator regression; SVM, support vector machine; LSVM, linear SVM; Ridge, ridge regression; FNN, feed-forward neural network.

Random forests were seen to be the most predictive model in some of the sets, but also often failed to improve over the gBLUP, making it hard to strongly recommend. Convolutional neural networks also tended to have good predictive performance except in the cases where there was clear failure on one of the cross-validation sets due to model misspecification (see Figs. 2a, 3d). This instability in prediction is an important observation and would not have been seen if not for the nested design. For this reason, in future model-building, it would be recommended to look closely for full robustness on any validation sets before moving to a final test set.

Two overall trends are also clear from the plots: that using a larger SNP-set size leads to improved performance; and that feature selection through linear association (GWAS) also aids in decreasing predictive error.

The statistical comparisons between models for the other two traits, seed yield and leaf area, can be seen in Fig. 4. Unlike with the flowering traits, none of the machine learning models was able to significantly outperform the baseline gBLUP method. In fact, some of the models significantly underperformed, particularly LASSO regression.

Discussion

The results presented here show the potential for non-linear models to improve upon linear genomic prediction in *Arabidopsis*. The possibility for substantial improvement is worth pursuing given the struggles in the field to close the missing heritability gap

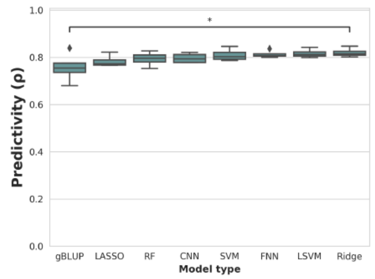

(a) Top 10,000 GWAS SNPs (clumped). Mean R^2 of gBLUP model is 0.58.

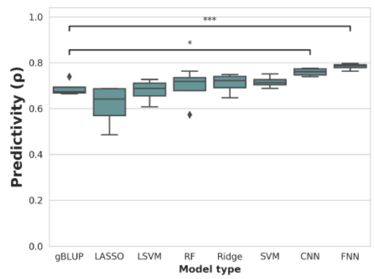

(b) Random 10,000 SNPs (pruned). Mean R^2 of gBLUP model is 0.47.

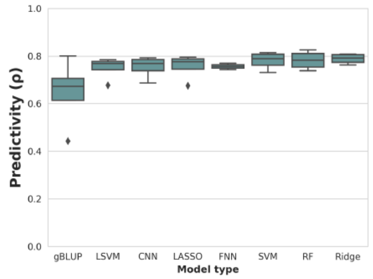

(c) Top 1,000 GWAS SNPs (clumped). Mean R^2 of gBLUP model is 0.44.

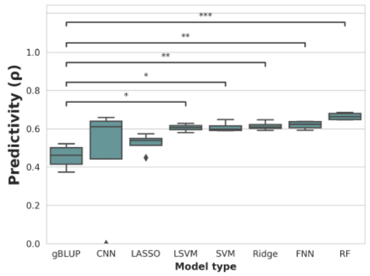

(d) Random 1,000 SNPs (pruned). Mean R^2 of gBLUP model is 0.21.

Fig 3. Nested cross-validation results for flowering time (16°C). Models are sorted by mean performance. Dunnett test p-value annotations: $*, p < 0.05$; $**, p < 0.01$; $***, p < 0.001$. gBLUP, genomic best linear unbiased prediction; CNN, convolutional neural network; RF, random forests; LASSO, least absolute shrinkage and selection operator regression; SVM, support vector machine; LSVM, linear SVM; Ridge, ridge regression; FNN, feed-forward neural network.

despite large sample sizes and dense marker sets. Improvements in prediction were dependant on model type, feature set size, feature selection method, and trait.

Overall, the baseline gBLUP model yielded reasonable predictions in comparison to the machine learning models, a not unexpected result from prior research. However, for flowering traits, some machine learning families could be seen to perform consistently better than the baseline model, such as neural networks and ridge regression.

This improvement in performance was often significant, despite the lower statistical power the cross-validation design gave for performance assessment. Several previous studies on machine learning in genomic prediction have omitted statistical tests, presumably on the basis of low power, although it is an important component of assessing improvements in performance.

For the other two traits, none of the machine learning models was able to significantly outperform the baseline model. These traits had lower heritabilities and smaller sample sizes than flowering time which could be contributing factors to this result. This adds to the existing body of evidence that improvement in performance using machine learning is not guaranteed and is highly dependant on trait and dataset.

Performance in prediction generally improved when using more SNP markers. Despite a much smaller number of individuals than predictors, overfitting with so many variables did not seem to be an issue, as might happen in $P \gg N$ problems.

Additionally, prior linear association for feature selection was found to be a useful step in this study, which is not guaranteed when modelling non-additive interactions [42, 43]; this performance boost was not extreme, however.

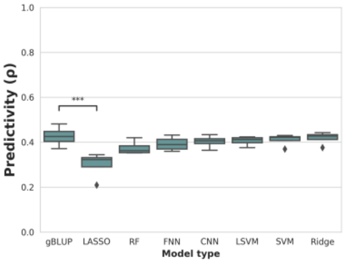

(a) Top 10,000 GWAS SNPs (clumped) of leaf area. Mean R^2 of gBLUP model is 0.18.

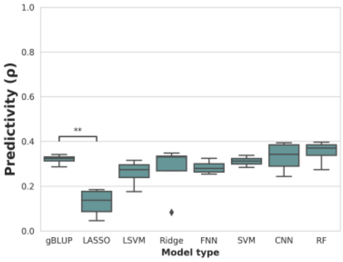

(b) Random 10,000 SNPs (pruned) of leaf area. Mean R^2 of gBLUP model is 0.10.

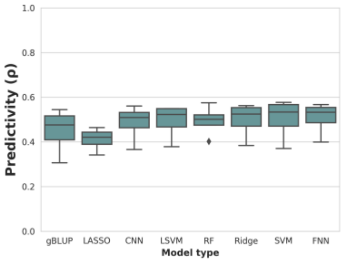

(c) Top 10,000 SNPs (clumped) of seed yield. Mean R^2 of gBLUP model is 0.21.

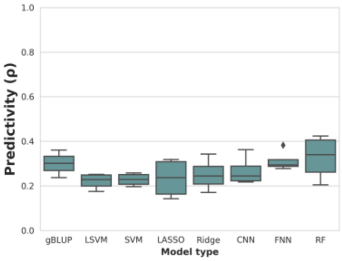

(d) Random 10,000 SNPs (pruned) of seed yield. Mean R^2 of gBLUP model is 0.09.

Fig 4. Nested Cross-Validation Results for seed yield and leaf area. Models are sorted by mean performance. Dunnett test p-value annotations: $*, p < 0.05$; $**, p < 0.01$; $***, p < 0.001$. gBLUP, genomic best linear unbiased prediction; CNN, convolutional neural network; RF, random forests; LASSO, least absolute shrinkage and selection operator regression; SVM, support vector machine; LSVM, linear SVM; Ridge, ridge regression; FNN, feed-forward neural network.

Interestingly, the performance improvement for the best non-linear models over the linear models was more pronounced when using random SNPs, which, unlike the top GWAS SNPs, were not chosen for their prior linear association. It is perhaps understandable that the most linearly associated features tend to explain the most variance when combined in a linear fashion; however, it is evident that a substantial amount of variation stands to be captured from the rest of the genome, where non-linear effects may play a larger role [44, 45]. For this reason, it is often noted, and this study reiterates, that linear feature selection may overlook important variables that combine non-linearly.

Notwithstanding this possibility, due to computational limitations, a subset of SNPs had to be chosen as input in this study and it was found from this study that the optimal input features were the most significant QTLs obtained from a GWAS. As compute power grows, however, one should remain open-minded as to the input search space when using genotypic information to predict complex traits.

For example, it is possible that widespread biological and statistical epistasis exists for these traits which is why the non-linear models were sometimes successful in explaining more trait heritability. Therefore, a better understanding of the total genetic architecture of a trait could aid in building more powerful models [9]. One of the drawbacks of machine learning, however, is that the models are often "black boxes" that are not readily interpretable [15]. Although improved prediction can hint towards a greater role for epistasis in the variance of complex traits, elucidating the specific

complex interactions is not a simple task. One possible extension of this study would be to find putative epistatic interactions through linear methods in the feature selection step.

Genomic prediction is concerned not just with performance, but also with ensuring that methods and models are robust enough to be reliable, consistent and transferable across datasets. This study used a nested-cross validation approach for hyperparameter tuning in order to assure the reliability of the results. This approach was useful in identifying a pitfall of hyperparameter tuning using k-fold cross-validation, namely the potential to miss serious misspecification on the final models despite success on validation sets.

Limitations of this analysis include the modest sample sizes for the various traits and the lack of a fully independent external test dataset. In the field, there would also be a larger non-genetic component of variation, as well as genotype-environment interactions, which could possibly affect the relative performances of the various machine learning models. Furthermore, the individuals in this analysis were inbred to homozygosity which simplified the input genotype matrices and removed intra-allelic effects such as dominance. Any of these considerations could cause a difference in model performance in natural populations [46].

As the feature selection step was linear in this analysis, the GRM employed in the MLMA should have somewhat mitigated the effect of population structure, yet the issue of confounding is a central one in genomic prediction and it not easily addressed here.

Firstly, in the field of machine learning, the most appropriate methods for handling confounding variables which are often needed in genetic studies, such as through using the first principal components of variation as linear covariates in a regression, have not been well established [47–49].

Secondly, the individuals in this analysis were grown under standardized laboratory conditions which theoretically removes the effect of confounding by means of a correlation between genotype and environmental exposure (e.g. differences in soil type and mineral concentrations). However, to the extent that there is correlation between ancestry informative SNPs and causal loci (and ignoring genotype-environment interactions), fully accounting for this population structure may reduce predictive performance in such a way as to be undesirable [50, 51]. As the final model would ultimately be predicting phenotype from genotype, such information should not necessarily be excluded when trying to maximize the explained variance across real populations in potentially varied environments.

Thirdly, although many machine learning models are black boxes in terms of causal inference, it might still be an important goal of a prediction tool to identify relevant genes, variants and pathways that are being used by the model.

For the above stated reasons, we have left ascertaining the best approach to handling confounding and covariate adjustment in using machine learning for genomic prediction to future work on separate datasets.

Supporting information

S1 Grid Search Parameters.

Feed-forward neural network: Number of units, learning rate, optimizer, batch size, network shape, L1/L2 regularization, dropout rate, number of hidden layers, epochs, kernel initializer, activation function

Convolutional neural network: Number of units, learning rate, optimizer, batch size, network shape, L1/L2 regularization, dropout rate, number of hidden layers, epochs, kernel initializer, activation function, filters, kernel, pool, strides

SVM: C, gamma, epsilon, loss, kernel, degree, cache size, tolerance

LASSO: Alpha, selection, tolerance, maximum iterations

Ridge regression: Alpha, tolerance

Random forests: Number of estimators, maximum depth, maximum number of features, bootstrapping, maximum leaf nodes, maximum number of samples, minimum samples to split, minimum leaf samples, maximum leaf nodes

Acknowledgments

Many thanks to Ross Byrne, Mark Doherty, Jennifer Hengeveld, Laura O'Briain, the Bradley lab and the Cassidy lab.

References

1. Falconer DS, Mackay TFC. Introduction to Quantitative Genetics. 4th ed. Pearson Education; 1995.

2. Meuwissen THE, Hayes BJ, Goddard ME. Prediction of Total Genetic Value Using Genome-Wide Dense Marker Maps. Genetics. 2001;157(4):1819–1829. doi:10.1093/genetics/157.4.1819.

3. Moose SP, Mumm RH. Molecular Plant Breeding as the Foundation for 21st Century Crop Improvement. Plant Physiology. 2008;147(3):969–977. doi:10.1104/pp.108.118232.

4. Cordell HJ. Epistasis: what it means, what it doesn't mean, and statistical methods to detect it in humans. Human molecular genetics. 2002;11(20):2463–8. doi:10.1093/hmg/11.20.2463.

5. Brachi B, Morris GP, Borevitz JO. Genome-wide association studies in plants: the missing heritability is in the field. Genome Biology. 2011;12(10):232. doi:10.1186/gb-2011-12-10-232.

6. Mäki-Tanila A, Hill WG. Influence of Gene Interaction on Complex Trait Variation with Multilocus Models. Genetics. 2014;198(1):355–367. doi:10.1534/genetics.114.165282.

7. Zhu Z, Bakshi A, Vinkhuyzen AAE, Hemani G, Lee SH, Nolte IM, et al. Dominance Genetic Variation Contributes Little to the Missing Heritability for Human Complex Traits. The American Journal of Human Genetics. 2015;96(3):377–385. doi:10.1016/j.ajhg.2015.01.001.

8. Huang W, Mackay TFC. The Genetic Architecture of Quantitative Traits Cannot Be Inferred from Variance Component Analysis. PLOS Genetics. 2016;12(11):e1006421. doi:10.1371/journal.pgen.1006421.

9. Ober U, Huang W, Magwire M, Schlather M, Simianer H, Mackay TFC. Accounting for genetic architecture improves sequence based genomic prediction for a Drosophila fitness trait. PloS one. 2015;10(5):e0126880. doi:10.1371/journal.pone.0126880.

10. Dai Z, Long N, Huang W. Influence of Genetic Interactions on Polygenic Prediction. G3 Genes—Genomes—Genetics. 2020;10(1):109–115. doi:10.1534/g3.119.400812.

11. Gupta PK, Kulwal PL, Jaiswal V. Chapter Two - Association mapping in plants in the post-GWAS genomics era. vol. 104 of Advances in Genetics. Academic Press; 2019. p. 75–154. Available from: https://www.sciencedirect.com/science/article/pii/S0065266018300385.

12. Habier D, Fernando RL, Dekkers JCM. The Impact of Genetic Relationship Information on Genome-Assisted Breeding Values. Genetics. 2007;177(4):2389–2397. doi:10.1534/genetics.107.081190.

13. VanRaden PM. Efficient Methods to Compute Genomic Predictions. Journal of Dairy Science. 2008;91(11):4414–4423. doi:10.3168/jds.2007-0980.

14. Yang J, Benyamin B, McEvoy BP, Gordon S, Henders AK, Nyholt DR, et al. Common SNPs explain a large proportion of the heritability for human height. Nature Genetics. 2010;42(7):565–569. doi:10.1038/ng.608.

15. Friedman J, Hastie T, Tibshirani R. The Elements of Statistical Learning. 2nd ed. New York: Springer Science & Business Media; 2009.

16. Azodi CB, Bolger E, McCarren A, Roantree M, de los Campos G, Shiu SH. Benchmarking Parametric and Machine Learning Models for Genomic Prediction of Complex Traits. G3 Genes—Genomes—Genetics. 2019;9(11):3691–3702. doi:10.1534/g3.119.400498.

17. Grinberg NF, Lovatt A, Hegarty M, Lovatt A, Skøt KP, Kelly R, et al. Implementation of Genomic Prediction in Lolium perenne (L.) Breeding Populations. Frontiers in Plant Science. 2016;7. doi:10.3389/fpls.2016.00133.

18. Toda Y, Wakatsuki H, Aoike T, Kajiya-Kanegae H, Yamasaki M, Yoshioka T, et al. Predicting biomass of rice with intermediate traits: Modeling method combining crop growth models and genomic prediction models. PLOS ONE. 2020;15(6):e0233951. doi:10.1371/journal.pone.0233951.

19. Zingaretti LM, Gezan SA, Ferrão LFV, Osorio LF, Monfort A, Muñoz PR, et al. Exploring Deep Learning for Complex Trait Genomic Prediction in Polyploid Outcrossing Species. Frontiers in Plant Science. 2020;11. doi:10.3389/fpls.2020.00025.

20. van Dijk ADJ, Kootstra G, Kruijer W, de Ridder D. Machine learning in plant science and plant breeding. iScience. 2021;24(1):101890. doi:10.1016/j.isci.2020.101890.

21. Spiess AN, Neumeyer N. An evaluation of R^2 as an inadequate measure for nonlinear models in pharmacological and biochemical research: a Monte Carlo approach. BMC Pharmacology. 2010;10(1):6. doi:10.1186/1471-2210-10-6.

22. Sapra RL. Using R^2 with caution. Current Medicine Research and Practice. 2014;4(3):130–134. doi:10.1016/j.cmrp.2014.06.002.

23. Kvålseth TO. Cautionary note about R^2. The American Statistician. 1985;39(4):279–285. doi:10.1080/00031305.1985.10479448.

24. Tong H, Nikoloski Z. Machine learning approaches for crop improvement: Leveraging phenotypic and genotypic big data. Journal of Plant Physiology. 2021;257:153354. doi:10.1016/j.jplph.2020.153354.

25. Varma S, Simon R. Bias in error estimation when using cross-validation for model selection. BMC Bioinformatics. 2006;7(1):91. doi:10.1186/1471-2105-7-91.

26. Krstajic D, Buturovic LJ, Leahy DE, Thomas S. Cross-validation pitfalls when selecting and assessing regression and classification models. Journal of Cheminformatics. 2014;6(1):10. doi:10.1186/1758-2946-6-10.

27. Vabalas A, Gowen E, Poliakoff E, Casson AJ. Machine learning algorithm validation with a limited sample size. PLOS ONE. 2019;14(11):e0224365. doi:10.1371/journal.pone.0224365.

28. Bracher-Smith M, Crawford K, Escott-Price V. Machine learning for genetic prediction of psychiatric disorders: a systematic review. Molecular Psychiatry. 2021;26(1):70–79. doi:10.1038/s41380-020-0825-2.

29. Alonso-Blanco C, Andrade J, Becker C, Bemm F, Bergelson J, Borgwardt KM, et al. 1,135 Genomes Reveal the Global Pattern of Polymorphism in Arabidopsis thaliana. Cell. 2016;166(2):481–491. doi:10.1016/j.cell.2016.05.063.

30. Evans LM, Tahmasbi R, Vrieze SI, Abecasis GR, Das S, Gazal S, et al. Comparison of methods that use whole genome data to estimate the heritability and genetic architecture of complex traits. Nature Genetics. 2018;50(5):737–745. doi:10.1038/s41588-018-0108-x.

31. Purcell S, Neale B, Todd-Brown K, Thomas L, Ferreira MAR, Bender D, et al. PLINK: A Tool Set for Whole-Genome Association and Population-Based Linkage Analyses. The American Journal of Human Genetics. 2007;81(3):559–575. doi:10.1086/519795.

32. Chang CC, Chow CC, Tellier LC, Vattikuti S, Purcell SM, Lee JJ. Second-generation PLINK: rising to the challenge of larger and richer datasets. GigaScience. 2015;4(1):7. doi:10.1186/s13742-015-0047-8.

33. Pedregosa F, Varoquaux G, Gramfort A, Michel V, Thirion B, Grisel O, et al. Scikit-learn: Machine Learning in Python. Journal of Machine Learning Research. 2011;12:2825–2830.

34. Yang J, Zaitlen NA, Goddard ME, Visscher PM, Price AL. Advantages and pitfalls in the application of mixed-model association methods. Nature Genetics. 2014;46(2):100–6. doi:10.1038/ng.2876.

35. Chollet F, et al. Keras: The Python Deep Learning library. Available from: https://githubcom/fchollet/keras. 2015;.

36. Abadi M, Barham P, Chen J, Chen Z, Davis A, Dean J, et al. TensorFlow: A system for large-scale machine learning. In: Proceedings of the 12th USENIX Symposium on Operating Systems Design and Implementation, OSDI 2016; 2016.

37. Japkowicz N, Shah M. Evaluating Learning Algorithims: A Classification Perspective. New York: Cambridge University Press; 2011.

38. Maxwell SE, Delaney HD, Kelley K. Designing Experiments and Analyzing Data: A Model Comparison Perspective. 3rd ed. New York: Routledge; 2018.

39. Konietschke F, Placzek M, Schaarschmidt F, Hothorn LA. nparcomp: An R Software Package for Nonparametric Multiple Comparisons and Simultaneous Confidence Intervals. Journal of Statistical Software. 2015;64(9):1–17.

40. Hothorn T. Most Likely Transformations: The mlt Package. Journal of Statistical Software. 2020;92(1):1–68. doi:10.18637/jss.v092.i01.

41. Hothorn LA, Kluxen FM. Robust multiple comparisons against a control group with application in toxicology. arXiv:190501838v1 [statAP]. 2019;doi:10.48550/ARXIV.1905.01838.

42. Hua J, Tembe WD, Dougherty ER. Performance of feature-selection methods in the classification of high-dimension data. Pattern Recognition. 2009;42(3):409–424. doi:10.1016/j.patcog.2008.08.001.

43. Wolff RF, Moons KGM, Riley RD, Whiting PF, Westwood M, Collins GS, et al. PROBAST: A Tool to Assess the Risk of Bias and Applicability of Prediction Model Studies. Annals of Internal Medicine. 2019;170(1):51. doi:10.7326/M18-1376.

44. Urbanowicz RJ, Meeker M, La Cava W, Olson RS, Moore JH. Relief-based feature selection: Introduction and review. Journal of Biomedical Informatics. 2018;85:189–203. doi:10.1016/j.jbi.2018.07.014.

45. Krakovska O, Christie G, Sixsmith A, Ester M, Moreno S. Performance comparison of linear and non-linear feature selection methods for the analysis of large survey datasets. PloS one. 2019;14(3):e0213584. doi:10.1371/journal.pone.0213584.

46. Charmantier A, Garant D, Kruuk LEB. Quantitative Genetics in the Wild. Oxford: Oxford University Press; 2014. Available from: `https://oxford.universitypressscholarship.com/10.1093/acprof:oso/9780199674237.001.0001/acprof-9780199674237`.

47. Li L, Rakitsch B, Borgwardt K. ccSVM: correcting Support Vector Machines for confounding factors in biological data classification. Bioinformatics. 2011;27(13):i342–i348. doi:10.1093/bioinformatics/btr204.

48. He J, Baxter SL, Xu J, Xu J, Zhou X, Zhang K. The practical implementation of artificial intelligence technologies in medicine. Nature Medicine. 2019;25(1):30–36. doi:10.1038/s41591-018-0307-0.

49. Whalen S, Schreiber J, Noble WS, Pollard KS. Navigating the pitfalls of applying machine learning in genomics. Nature Reviews Genetics. 2022;23(3):169–181. doi:10.1038/s41576-021-00434-9.

50. Wray NR, Yang J, Hayes BJ, Price AL, Goddard ME, Visscher PM. Pitfalls of predicting complex traits from SNPs. Nature Reviews Genetics. 2013;14(7):507–515. doi:10.1038/nrg3457.

51. Barton N, Hermisson J, Nordborg M. Population Genetics: Why Structure Matters. eLife. 2019;8. doi:10.7554/eLife.45380.
