# Supplementary material for: Comparison of machine learning methods for genomic prediction of selected Arabidopsis thaliana traits
Source: PLoS One. 2024 Aug 28;19(8):e0308962. doi: 10.1371/journal.pone.0308962 (PMC11355539; doi:10.1371/journal.pone.0308962)
Supplement: Attachment — Submitted filename: comments.docx [file pone.0308962.s004.docx]

**Major Comments:**

1- The data is not provided in the github repository and therefore, scripts are not working. Moreover, I could not find scripts of ML methods.
2- Population structure and data specifics are not discussed. Also, population structure is not incorporated (though discussed), so the predictive abilities can be overestimated. I would suggest incorporating it in any case for comparison or show the specifics of the genotype data, if it is not significant.
3- GBLUP and Ridge are showing different performances. However, literature suggests their equivalence (Meuwissen et al. 2001).
4- I would appreciate to see the outcomes with full genotype dataset.
5- Pruning using linear methods restricts it to the SNPs with main effects. This would remove any SNPs with interactions and having minor main effects. For this purpose, I would suggest including some non-linear feature selector.
6- For a high heritability, we can expect the model to make minimal errors and the accuracy should achieve theoretical maximum. In your case, it saturates to ~0.8, which is slightly lower. Would be please explain it, why?

**Minor Comments:**

1- I would like to see why 1001 genome project’s population would be a good to test GP models and how would it be consistent with a more realistic breeding population.
2- In the abstract and lines 12-14, it was mentioned that linear models may be impeded by their inability to make use of non-additive effects. This may not be true because many linear models can accommodate non-additive effects. For instance, EGBLUP by Martini et al., 2017
3- In the abstract, it was mentioned that developing complex models with larger SNP sets can improve performance. I don't think it is right, because complex models tend to be overfitted as well.
4- The introduction section doesn't connect this work to some earlier studies on GP using *A. thaliana* very well. For instance, a recent work by Farooq et al., 2022 etc. on this topic.
5- Only three traits were explored but the conclusions and the title/abstract seem overambitious. May be narrowing down the scope to these three traits and explicitly mentioning this in the title / abstract would help the reader.
6- Line 42 says GBLUP uses fixed effects for SNPs, whereas, it uses random effects and solves SNP effects through mixed model’s equations.
7- Line 69-70 says that all ML methods are non-linear, whereas, Ridge and Lasso are linear.
8- Lines 90-92: please explain why only these methods were chosen?
9- I don't think figure 1 adds much information to the main text. It can be in the supplementary.
10- Line 136: A. thaliana has 5 chromosomes, whereas, you mentioned it as four.
11- Line 205: you mentioned that non-genetic variance was constrained by the laboratory conditions. Yes, that can be the case, but we should still expect epistasis and dominance, though, GxE is limited.
