# Supplementary material for: Comparison of machine learning methods for genomic prediction of selected Arabidopsis thaliana traits
Source: PLoS One. 2024 Aug 28;19(8):e0308962. doi: 10.1371/journal.pone.0308962 (PMC11355539; doi:10.1371/journal.pone.0308962)
Supplement: Attachment — Submitted filename: Response to Reviewers.docx [file pone.0308962.s005.docx]

**Additional Editor Comments:**

Thank you for your submission. We received two reviews of your work, and I also reviewed the article. Please address all of the reviewers’ concerns.

A number of reviewers’ concerns were shared. Both reviewers would like to see the code and data set made available. Both reviewers were concerned that the article does not sufficiently explain its contribution to science. Finally, both reviewers were concerned with model accuracy and how population structure affected it.

The authors greatly appreciate the time taken by the reviewers and the editors with the manuscript. Consideration was given to all points made and changed to the manuscript have been made as per the comments below. We hope that the comments and corrected manuscript have addressed all concerns in moving forward with the article.

All code should now be publicly available on GitHub (<https://github.com/ciaranoceallaigh96/arabidopsis_ml/>). Please let us know if there are any issues. More explicit links to the genotype and phenotype data have been given.

With regards to contributions to science, we believe that this contributes to the work on the open question as to if, and when, machine learning models can out-compete standard genomic prediction methods. It attempts to do so in a robust, statistical manner. Although some similar works have been published elsewhere, if these specific results, which are non-significant for some traits, were not disseminated this could contribute to publication bias on this topic. For example, if this dataset showed machine learning models greatly outperforming the linear models, it might be seen as more novel work and therefore more likely to contribute to science. This would of course skew the record in favour of machine learning models. This work attempts to be as robust as possible in statistically comparing between models, using nested-cross validation during evaluation of model performance.

The issue of population structure in genomic prediction is a profound one, and is discussed again below as per the reviewer comments. There are robust approaches to tackling the confounding as mediated by population structure in traditional linear settings such as through the use of LMMs. The LMM/MLMA approach is employed in this work to guard against some of the effects of population structure. However, the extent to which they can prevent confounding in a machine-learning setting is unclear. There is no current gold-standard method for addressing confounding in non-linear frameworks. Perhaps the greatest issue of population structure as it relates to genomic prediction is confounding by environmental effects, however, this form of confounding is not present in this dataset due to the standardized laboratory conditions. Overall, we agree that this is a limitation, which the manuscript notes. The most fruitful work on this issue will most likely be done using natural datasets with known environmental confounders that can be measured and tracked. We believe this question is not best addressed by this dataset, and that measuring the success of fully controlling for all effects population structure is not yet possible (and perhaps desirable) in this setting.

My points also overlap with these concerns. First, the article’s premise should be clarified. The idea seems to be that additive linear models do not capture non-additive interactions among loci, so machine learning methods should be applied for prediction. However, linear models do capture epistasis. As one empirical example, we have shown that linear model analysis of Arabidopsis progeny whose traits are fully explained by two-gene epistasis will nonetheless assign additive effects to parental lines. Since additive models capture variance due to epistasis, the models may predict genotypic values quite well, but the genotypic values may not predict offspring values well (e.g. see line 11).

The article makes the point that indeed linear models can capture some epistatic effects in lines 16-19 of the original manuscript. However, that does not imply that linear models can perfectly exploit non-linear interactions to their full extent in individual-level prediction, as is stated in lines 24-31 and the references therein. A purely linear model cannot account for the entirety of the broad-sense heritability of a complex trait, as it is bounded by the narrow-sense heritability in explanatory power. If the goal is completely accurate prediction in previously unseen individuals, then it is likely that non-linear models will be utilized (if the complexity behind quantitative trait values can indeed by modelled appropriately).

A second issue is that the manuscript can’t ascribe performance differences between machine learning methods and linear models to ML abilities to capture epistasis. Methods predictions vary for unknown reasons. Some ML methods have better predictive abilities than additive models and some worse. I would remove text from the abstract, introduction, and elsewhere that attributes differences in models’ performances to known genetic phenomena (e.g. paragraphs 2-4).

We absolutely agree with this important point and have noted this in lines 284-289. This is a fair criticism and some of the stronger language on this point has been attenuated. It is unlikely that, if machine learning models are to substantially improve upon predictive performance in the next decade, this increase in performance will explicitly be shown to be due to modelling epistasis (although this might be expected to be true). Predictive accuracy is likely to increase a lot faster than our understanding of the causal pathways behind models.

Second, I am not very familiar with the 1001 genome project, but given the lines are from different latitudes and altitudes, population structure is causing the marker-flowering time associations. As a result, the paper’s objective seems to be to evaluate methods that best capture population differences. This does not seem useful. If one can predict a genotype’s flowering time based on latitude and altitude, why would one perform genomic predictions? It would be more interesting to predict traits after removing population structure. Even removing the effects of each entry’s latitude and altitude collection site may have a major effect on results.

The issue of population structure is indeed of serious importance (as per lines 306-322), but can not be fully addressed here for two reasons (i) As all strains were grown in standardized conditions where there can be no confounding by environmental factors correlated with genetic structure, as in wild populations. There may indeed by confounding by the shared genetic background (this is noted), but this likely is a more serious problem for association of single loci and the elucidation of causal pathways, rather than overall accuracy of prediction. As is stated in lines 316-322 (and related references), it is not always desirable to fully control for this form of confounding if the ultimate goal is predictive accuracy (ii) there is no gold-standard approach for accounting for population structure in non-linear machine-learning approaches to genomic prediction. The standard linear approaches (e.g. PCA) will not apply in these contexts. There is important work to be done on this issue, but it is likely that in future several different methods, with several different benchmarks, will be proposed. For this reason, it was considered beyond the scope of the article to fully addressed, although using MLMA with a GRM should in principle account for confounding in choosing the input SNPs (lines 306-308). 

Finally, predictions would likely be improved by using more SNPs. Why were 1,000 or 10,000 SNPs used? This seems very low, and I suspect many more could be used without running into “computational limitations.” To look into this, I’d plot the number of markers and their predictions for different methods. Even if there are computational limitations with some approaches and not others, it is still worth knowing the best predictions you can get with the all data at hand.

The manuscript does attempt to address this issue by comparing SNP-set sizes that were achievable. A maximum of 10,000 SNPs were used for computational and time limitations within the nested-cross validation framework with extensive hyperparameter tuning (especially for random forests and neural networks). It is simply not possible using the described methods to include many more SNPs in a reasonable timeframe, given the approach taken and resources available.

I found a few sentences and topics confusing. For example, the lines were grown in one experimental condition. Because there is no G x E, I did not understand why it is discussed at the end of the manuscript.

This manuscript has been edited to clarify this point. The issue of GxE is mentioned in lines 299-305, as a limitation of the analysis. As the standardized conditions do not reflect real-life conditions, this is a limitation of the dataset and may affect generalization of the results in other settings. This point could be removed, but it is thought to be important to highlight to a reader, who may not be explicitly aware of this consideration.


[Note: HTML markup is below. Please do not edit.]

Reviewers' comments:

Reviewer's Responses to Questions

**Comments to the Author**

1. Is the manuscript technically sound, and do the data support the conclusions?

The manuscript must describe a technically sound piece of scientific research with data that supports the conclusions. Experiments must have been conducted rigorously, with appropriate controls, replication, and sample sizes. The conclusions must be drawn appropriately based on the data presented.

Reviewer #1: Partly

Reviewer #2: Partly

2. Has the statistical analysis been performed appropriately and rigorously?

Reviewer #1: Yes

Reviewer #2: Yes

3. Have the authors made all data underlying the findings in their manuscript fully available?

The [PLOS Data policy](http://www.plosone.org/static/policies.action#sharing) requires authors to make all data underlying the findings described in their manuscript fully available without restriction, with rare exception (please refer to the Data Availability Statement in the manuscript PDF file). The data should be provided as part of the manuscript or its supporting information, or deposited to a public repository. For example, in addition to summary statistics, the data points behind means, medians and variance measures should be available. If there are restrictions on publicly sharing data—e.g. participant privacy or use of data from a third party—those must be specified.

Reviewer #1: No

Reviewer #2: No

4. Is the manuscript presented in an intelligible fashion and written in standard English?

PLOS ONE does not copyedit accepted manuscripts, so the language in submitted articles must be clear, correct, and unambiguous. Any typographical or grammatical errors should be corrected at revision, so please note any specific errors here.

Reviewer #1: Yes

Reviewer #2: Yes

5. Review Comments to the Author

Please use the space provided to explain your answers to the questions above. You may also include additional comments for the author, including concerns about dual publication, research ethics, or publication ethics. (Please upload your review as an attachment if it exceeds 20,000 characters)

Reviewer #1: The manuscript by Kelly and McLaughlin presents an analysis of using various Machine Learning (ML) algorithms to perform genomic prediction, and compares the performance of these methods with the more traditional gBLUP approach. Such comparison could be quite useful, but recently various authors have presented similar results which are not cited in the manuscript (e.g. <https://www.ncbi.nlm.nih.gov/pmc/articles/PMC10080209/>; <https://pubmed.ncbi.nlm.nih.gov/34792168>; <https://www.frontiersin.org/articles/10.3389/fpls.2022.932512/full>; and various others). It would be good to describe more comprehensively what was already learned in these existing publications.

Lines 82-85 make reference to previous similar work in a plant context, but the manuscript has been updated to make more specific reference to the work suggested. Raimondi et al. (2022) tests only neural networks and, whilst it does find flowering time to be amenable to prediction by them, it is not a comparison between various different methods. A strength of our submitted analysis is its comparison between several methods within the same statistical tests and the same baseline model. This may be useful for researchers unsure of which algorithms to explore in future work. Farooq et al. (2023), use a different dataset (RegMap), and their work is an important reference point that is now included. John et al. (2022) is similar to our study in principle; however, they evaluate models using explained variance which is not appropriate when comparing non-linear models (see lines 96-103 and the references therein). Furthermore, whilst John et al. (2022) use the same dataset, they do not use all same phenotypes.

This leads to my major concern:
(1) What does this manuscript add to the results presented in Raimondi et al. (2022) [<https://pubmed.ncbi.nlm.nih.gov/34792168/>]. In that paper, much more traits are used from the same Arabidopsis dataset as used in the current manuscript; it is also shown, similar as in the current manuscript, that neural networks can do well; and it is also shown, similar as in the current manuscript, that flowering time is one of the type of traits which are predicted best. It would be important to refer to this study and also make more clear what is done differently in the current work in order to obtain useful additional results, and to describe what insights are obtained that are not available in Raimondi et al.

References to Raimondi et al. (2022) are now included. As above, Raimondi et al. (2022) test only neural networks and, whilst it does find flowering time to be amenable to prediction by them, it is not a comparison between various different methods. A strength of this analysis is to compare several methods within the same statistical tests and the same baseline model. This may be useful for researchers unsure of which algorithms to explore in future work.

(2) In any case, it is unclear why the four specific traits which are used in the manuscript, were chosen out of the larger set of traits available in the Arabidopsis dataset which is used. Some argumentation for this would be needed, or better, a more extensive analysis of the different traits would be warranted.

Lines 138-140 state that flowering time trait is a quintessential *Arabidopsis* quantitative trait and had the largest available sample size. The manuscript has been updated to highlight the reasoning for the other traits. They were chosen simply as the traits that had the largest sample size available, which is critical for robust nested-cross validation.

(3) Code used for training the machine learning models does not seem available.

Should now be available (see response to editor).

In addition, some minor comments:
(4) How about relatedness between the individuals - how does this influence prediction performance? Should this not be taken into account in making training-test splits (to prevent that out of two very related individuals, one is in the training set and the other in the test set)?

The geographic spread of the samples is wide, although it is true that certain groups may be closely related, such as North American samples. Inclusion of the GRM during the MLMA should have mitigated some of the unwanted effects of relatedness. As per the original protocol for analysis in *The 1001 Genomes Consortium* (see 1,135 Genomes Reveal the Global Pattern of Polymorphism in Arabidopsis thaliana) all accessions collected and genotyped for the project were chosen to be included in the study.

(5) Two sentences in the methods could be further clarified (p.6): “Phenotype values were standardized and scaled based on the training set prior to learning"; and "Random grid searching and manual evaluation on the inner loops was implemented to optimize the hyperparameters". It would be good to give additional details here on how this was done.

The manuscript has been updated to give more specific detail on these points. The phenotypes were standardized and scaled using *scikit-learn*’s StandardScaler() function. The performance for each value of the hyperparameter was box-plotted across the runs, with low-performing values removed in subsequent runs with a final-grid search on the subset of parameter values.

Reviewer #2: Major Comments:

1- The data is not provided in the github repository and therefore, scripts are not working. Moreover, I could not find scripts of ML methods.

Should now be available (see response to editor).

2- Population structure and data specifics are not discussed. Also, population structure is not incorporated (though discussed), so the predictive abilities can be overestimated. I would suggest incorporating it in any case for comparison or show the specifics of the genotype data, if it is not significant.

The issue of population structure is indeed of serious importance (as per lines 306-322), but can not be fully addressed here for two reasons (i) As all strains were grown in standardized conditions where there can be no confounding by environmental factors as mediated by population structure. There may indeed by confounding by the shared genetic background (this is noted), but this is a more serious problem for association of single loci and the elucidation of causal pathways, rather than overall accuracy of prediction. As is stated in lines 316-322 (and related references), it is not always desirable to fully control for this form of confounding if the ultimate goal is predictive accuracy (ii) there is no gold-standard approach for accounting for population structure in non-linear machine-learning approaches to genomic prediction. The standard linear approaches (e.g. PCA) will not apply in these contexts. There is important work to be done on this issue, but it is likely that in future several different methods, with several different benchmarks, will be proposed. For this reason, it was considered beyond the scope of the article to fully addressed, although using MLMA with a GRM should account for confounding in choosing the input SNPs (lines 306-308). 

3- GBLUP and Ridge are showing different performances. However, literature suggests their equivalence (Meuwissen et al. 2001).

It is true that ridge can be equivalent to GBLUP, but only under certain assumptions (e.g. with even QTL distribution across the genome) which are presumably not met here (see Bernardo 2020, https://doi.org/10.1038/s41437-020-0312-1).

4- I would appreciate to see the outcomes with full genotype dataset.

It is probably best to incorporate some LD-based pruning in the SNP-set selection. Furthermore, this would not be possible for time and computational reasons (see above/below).

5- Pruning using linear methods restricts it to the SNPs with main effects. This would remove any SNPs with interactions and having minor main effects. For this purpose, I would suggest including some non-linear feature selector.

The pruning step is only a function of LD patterns and not main effects. However, clumping does take the main effects into account. For this reason, the performance of the clumping approach is compared against the pruned set. However, there are of course limitations to this method, which is acknowledged in lines 267-274. A feature selector detecting epistatic effects is proposed as extensions to the work. One could possibly choose between a great many non-linear feature selectors, and it may be dependent on the underlying genetic architecture which method would yield the best result. It is unclear which non-linear feature selectors should be trialled. For this reason, it was thought prudent to try both not sub-setting by main effects, and sub-setting by the simplest linear approach (clumping), whilst acknowledging the limitations and opportunities.

6- For a high heritability, we can expect the model to make minimal errors and the accuracy should achieve theoretical maximum. In your case, it saturates to ~0.8, which is slightly lower. Would be please explain it, why?

It is hard to know why the model cannot explain the entirety of the genetic variation as measured by the heritability. It is possible that many factors e.g. more extensive hyperparameter tuning, more SNPs, less LD pruning, more complex modelling, increased sample size etc. could increase the variation explained.

Minor Comments:

1- I would like to see why 1001 genome project’s population would be a good to test GP models and how would it be consistent with a more realistic breeding population.

We accept that this is an important and reasonable point, and the limitations of not reflecting a real-world population are acknowledged in lines 299-305. Our main motivation for conducting these analyses with this dataset is that a more realistic breeding population would also bring along confounding by environmental effects, which is a problem that has yet to be solved in non-linear approaches to genomic prediction.

2- In the abstract and lines 12-14, it was mentioned that linear models may be impeded by their inability to make use of non-additive effects. This may not be true because many linear models can accommodate non-additive effects. For instance, EGBLUP by Martini et al., 2017

This point is addressed in the following paragraph of the manuscript. Whilst linear models can capture non-additive interaction, it is still true that there is a fundamental limitation to linear modelling for complex quantitative traits. Given the (presumed) genetic architecture of most complex traits, only a non-linear model would be able to fully explain the broad-sense heritability.

3- In the abstract, it was mentioned that developing complex models with larger SNP sets can improve performance. I don't think it is right, because complex models tend to be overfitted as well.

Over-fitting is a real issue, however, the abstract suggests larger sample sizes and modelling complexity, not SNP-set sizes, may lead to improvements. It is yet unclear to what extent any of those factors, may improve performance, but the abstract is simply trying to imply that further investigation is warranted.

4- The introduction section doesn't connect this work to some earlier studies on GP using A. thaliana very well. For instance, a recent work by Farooq et al., 2022 etc. on this topic.

As above, Farooq et al. (2023), use a different dataset (RegMap), but their work is an important reference point that is now included.

5- Only three traits were explored but the conclusions and the title/abstract seem overambitious. May be narrowing down the scope to these three traits and explicitly mentioning this in the title / abstract would help the reader.

This is a fair point and language has been changed in the abstract to reflect the narrower scope of the article.

6- Line 42 says GBLUP uses fixed effects for SNPs, whereas it uses random effects and solves SNP effects through mixed model’s equations.

This is correct and our error has been fixed in the manuscript.

7- Line 69-70 says that all ML methods are non-linear, whereas Ridge and Lasso are linear.

This may be simply a mis-reading, as lines 69-70 do state that Ridge and Lasso are linear.

8- Lines 90-92: please explain why only these methods were chosen.

The manuscript has been changed to reflect the reasoning, which is simply due to the popularity of the algorithms, so that the manuscript may serve as a reference point to interested researchers aiming to choose between these methods for their own genomic prediction tasks.

9- I don't think figure 1 adds much information to the main text. It can be in the supplementary.

Figure 1 has been moved to the supplementary section.
10- Line 136: A. thaliana has 5 chromosomes, whereas you mentioned it as four.

This was an error and has been fixed.

11- Line 205: you mentioned that non-genetic variance was constrained by the laboratory conditions. Yes, that can be the case, but we should still expect epistasis and dominance, though, GxE is limited.

This is indeed true, and the manuscript has been edited to make this point more explicitly clear. We thank the reviewer for pointing this out.

6. PLOS authors have the option to publish the peer review history of their article ([what does this mean?](https://journals.plos.org/plosone/s/editorial-and-peer-review-process#loc-peer-review-history)). If published, this will include your full peer review and any attached files.


If you choose “no”, your identity will remain anonymous but your review may still be made public.


**Do you want your identity to be public for this peer review?** For information about this choice, including consent withdrawal, please see our [Privacy Policy](https://www.plos.org/privacy-policy).

Reviewer #1: No

Reviewer #2: **Yes:** Muhammad Farooq (PhD)

---------------------------------------------------------------------------------------------------------------------------------------

--------------------------------------------------------------------------------------------------------------------------------------- ~~This study was supported by Science Foundation Ireland (17/CDA/4737). Russell~~

~~McLaughlin also receives support from the MND Association (879-791) and Science~~

~~Foundation Ireland~~

~~(16/RC/3948).~~

This study was supported by Science Foundation Ireland (17/CDA/4737). Russell McLaughlin also receives support from the Motor Neurone Disease Association (879-791) and Science Foundation Ireland (16/RC/3948). The funders had no role in study design, data collection and analysis, decision to publish, or preparation of the manuscript.

~~All genotypic and phenotypic data is available from the 1001 Arabidopsis Project~~

~~(~~[~~https://1001genomes.org/data-center.html~~](https://1001genomes.org/data-center.html)~~)~~

The genotypic data used in this study is available from the *1001 Arabidopsis Project* (https://1001genomes.org/data/GMI-MPI/releases/v3.1/SNP_matrix_imputed_hdf5/ 1001_SNP_MATRIX.tar.gz). The following phenotypic data was downloaded from the AraPheno database: flowering time at 10°C (<https://arapheno.1001genomes.org/phenotype/261/>), flowering time at 16°C (<https://arapheno.1001genomes.org/phenotype/262/>), leaf area (<https://arapheno.1001genomes.org/phenotype/108/>), seed yield (https://arapheno.1001genomes.org/phenotype/124/)
