# Supplementary material for: Comparison of machine learning methods for genomic prediction of selected Arabidopsis thaliana traits
Source: PLoS One. 2024 Aug 28;19(8):e0308962. doi: 10.1371/journal.pone.0308962 (PMC11355539; doi:10.1371/journal.pone.0308962)
Supplement: Attachment — Submitted filename: comments - R1.docx [file pone.0308962.s006.docx]

**Points still need to be addressed:**

1- The data is still not provided in the GitHub repository. Instead, it referred to the 1001 genome project page, which doesn’t allow it download straightforward. I would suggest it to be there in the git or the code should allow to reproduce the results by fetching it inside. The source code is still not fully reproducible. The article should not be online without having it.
2- Line 166-167: “Time to first flowering was chosen as the main trait of interest as the genetics of the trait has been extensively studied”. Please cite some references to show if it is well studied.
3- Line 198 and 203: different minimum allele counts were used. Please state why? Also, don’t you think it will create a bias for the comparison?
4- Predictions on all data are still missing (see last comments).
5- The order of model names should be consistent between figures on the horizontal axis for better readability and comparison. Also, the font sizes must be increased. Moreover, please mention how many data points of accuracies each boxplot represents. I have already suggested using multiple repeats of nested cross-validations for tackling PS.
