# Supplementary material for: Comparison of machine learning methods for genomic prediction of selected Arabidopsis thaliana traits
Source: PLoS One. 2024 Aug 28;19(8):e0308962. doi: 10.1371/journal.pone.0308962 (PMC11355539; doi:10.1371/journal.pone.0308962)
Supplement: Attachment — Submitted filename: Second Response to Reviewers.docx [file pone.0308962.s007.docx]

**Points still need to be addressed:**

1- The data is still not provided in the GitHub repository. Instead, it referred to the 1001 genome project page, which doesn’t allow it download straightforward. I would suggest it to be there in the git or the code should allow to reproduce the results by fetching it inside. The source code is still not fully reproducible. The article should not be online without having it.

The links to all genotype and phenotype downloads have now been given in the GitHub repository and the README has been updated to reflect this. We thank you for this comment.

2- Line 166-167: “Time to first flowering was chosen as the main trait of interest as the genetics of the trait has been extensively studied”. Please cite some references to show if it is well studied.

This is a fair point. We have now given references an interested can reader can follow-up on including a classic early paper exploring flowering time as a quantitative trait.

3- Line 198 and 203: different minimum allele counts were used. Please state why? Also, don’t you think it will create a bias for the comparison?

This was an omission on our part and thank the reviewer for the observation. The PLINK docs recommend a minimum MAC filter of 20 when using the GLM. “*The statistics computed by --glm are not calibrated well when the minor allele count is very small. --mac 20 is a reasonable filter to apply*” (see <https://www.cog-genomics.org/plink/2.0/assoc>). To the extent that it may or may not affect the results, it is a limitation of the statistical analysis used when employing PLINKS GLM that is not present in the other approach.

4- Predictions on all data are still missing (see last comments).

All predictions are now available on the GitHub repository at <https://github.com/ciaranoceallaigh96/arabidopsis_ml/blob/main/ml_predictions.zip> and <https://github.com/ciaranoceallaigh96/arabidopsis_ml/blob/main/pheno_and_gblup_predictions.zip> and the README has been updated to reflect this.

5- The order of model names should be consistent between figures on the horizontal axis for better readability and comparison. Also, the font sizes must be increased. Moreover, please mention how many data points of accuracies each boxplot represents. I have already suggested using multiple repeats of nested cross-validations for tackling PS.

As requested, we have changed the order of the model names and increased the font size as much as possible. We have also updated the figure legend to be clearer on the number of data points.
